# BRD4 promotes resection and homology-directed repair of DNA double-strand breaks

**DOI:** 10.1038/s41467-022-30787-6

**Published:** 2022-05-31

**Authors:** John K. Barrows, Baicheng Lin, Colleen E. Quaas, George Fullbright, Elizabeth N. Wallace, David T. Long

**Affiliations:** Department of Biochemistry and Molecular Biology, Medical University of South Carolina, Charleston, SC 29425 USA

**Keywords:** Homologous recombination, Chromatin remodelling

## Abstract

Double-strand breaks (DSBs) are one of the most toxic forms of DNA damage and represent a major source of genomic instability. Members of the bromodomain and extra-terminal (BET) protein family are characterized as epigenetic readers that regulate gene expression. However, evidence suggests that BET proteins also play a more direct role in DNA repair. Here, we establish a cell-free system using *Xenopus* egg extracts to elucidate the gene expression-independent functions of BET proteins in DSB repair. We identify the BET protein BRD4 as a critical regulator of homologous recombination and describe its role in stimulating DNA processing through interactions with the SWI/SNF chromatin remodeling complex and resection machinery. These results establish BRD4 as a multifunctional regulator of chromatin binding that links transcriptional activity and homology-directed repair.

## Abstract (web summary)

BRD4 is a multifunctional regulator of chromatin binding that plays a direct role in DNA double-strand break repair. BRD4 interacts with the SWI/SNF chromatin remodeling complex and resection machinery to promote homologous recombination.

## Introduction

Genomic DNA is frequently broken by both exogenous and endogenous agents^1,2^. In response, cells activate an adaptive DNA damage response that involves widespread epigenetic and gene expression changes. There are two major pathways of DSB repair: homologous recombination (HR) and non-homologous end joining (NHEJ)^3^. HR is favored during S and G2 phases due to the presence of a sister chromatid that provides a template for error-free repair^4^. In contrast, NHEJ is active throughout the cell cycle and involves direct ligation of DNA ends that can lead to insertions, deletions, or chromosomal translocations^5^. DSBs can also be repaired by alternative end joining (A-EJ) mechanisms like microhomology-mediated end joining^6^, where DNA ends are joined using small regions of homologous sequence near the break site^7^. Although many DNA repair pathways have been well characterized^2^, the highly integrated network of regulatory mechanisms that control competition between these different repair pathways remains poorly understood.

The bromodomain and extraterminal domain (BET) protein family has been shown to play an important role in regulating the cellular response to various forms of chromatin stress encountered during DNA replication, DNA repair, and telomere maintenance^8^. BET proteins (consisting of BRD2, BRD3, BRD4, and the testis-specific BRDT) are chromatin readers that recruit chromatin remodeling and transcription proteins to DNA through interactions with acetylated histone tails^9^. BET proteins have been found to regulate expression of various oncogenes^10–14^ and DNA repair factors^15–18^, identifying them as attractive targets for both single-agent and combinational anti-cancer therapies^19–21^. Reports have also linked BRD4 to R-loop maintenance^22^ and NHEJ^14,18^, suggesting it can play a more direct role in DNA repair. However, BRD4 has also been proposed to suppress DNA repair by promoting chromatin compaction^23,24^. Collectively, these studies reveal a critical but poorly understood role for BET proteins in DNA repair and genome maintenance.

Here, we use a new cell-free system to investigate the gene expression-independent functions of BET proteins in DSB repair. We show that this system supports multiple pathways of DSB repair that compete for access to DNA ends. We then specifically identify BRD4 as a critical regulator of repair that promotes DNA end processing through interactions with the SWI/SNF chromatin remodeling complex and resection machinery. Together with previous reports, our study helps to establish a comprehensive model that explains how BRD4 orchestrates repair pathway choice through a coordinated series of events. These results establish a gene expression-independent function for BRD4 that expands our understanding of its role in chromatin signaling and genome integrity.

## Results

### Competition between DSB repair pathways in *Xenopus* egg extracts

**Fig. 1:**
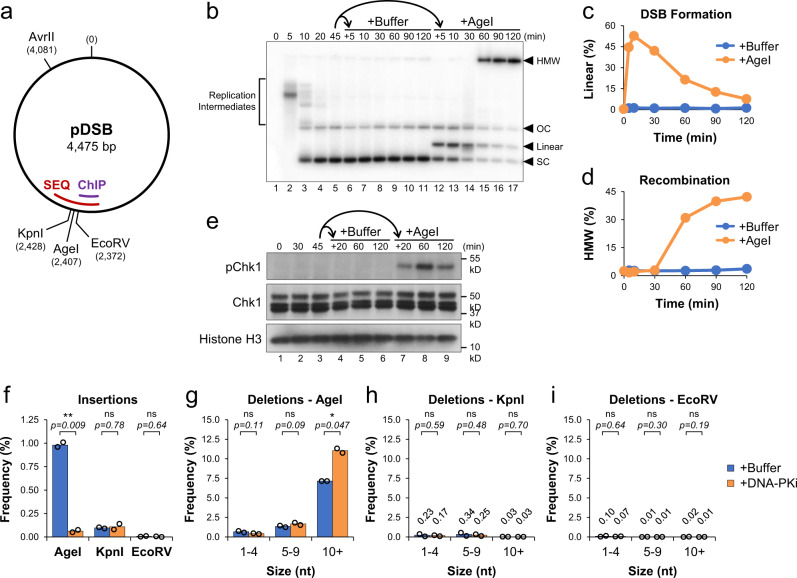
Competition between DSB repair pathways in *Xenopus* egg extracts. **a** Schematic of pDSB showing the relative position of restriction enzyme sites and DSB regions amplified for ChIP and amplicon sequencing (SEQ). **b** pDSB was replicated in the presence of α^32^P[dATP] for 45 min. The reaction was then split and supplemented with buffer or AgeI. Samples were withdrawn, resolved by 1D gel electrophoresis, and visualized by autoradiography (*n* = 4 independent experiments). Labels indicate the position of: replication intermediates, supercoiled (SC), linear, and open circular (OC) plasmids, and high molecular weight (HMW) molecules. **c**, **d** Quantitation of linear (**c**) and HMW (**d**) molecules from (**b**). **e** Protein samples from (**b**) were withdrawn and analyzed by Western blot (n = 2 independent experiments). **f**–**i** pDSB was replicated in extract supplemented with buffer or NU7441 (DNA-PKi) for 45 min. Reactions were then supplemented with AgeI, KpnI or EcoRV. Samples were withdrawn 30 min after enzyme addition and analyzed by amplicon sequencing (*n* = 2 independent experiments). Results are graphed to show the frequency of insertion products (**f**), and the frequency of different deletion products for AgeI, KpnI and EcoRV reactions (**g**–**i**). Data values are labeled for low frequency deletions in KpnI and EcoRV reactions. Student two-tailed t-test: not significant (ns), *p*-value < 0.05 (*), *p*-value < 0.01 (**).

The cellular response to DNA damage involves widespread changes in gene expression that make it difficult to distinguish between the direct and indirect functions of many proteins involved. To investigate the gene expression-independent mechanisms of DSB repair, we established a cell-free system using *Xenopus* egg extracts, which lack genomic DNA and mRNA translation^25^. We identified AgeI, KpnI, and EcoRV as restriction enzymes that readily cleave plasmid DNA in extract to produce DSBs with 5′ overhangs, 3′ overhangs, or blunt ends, respectively. A single plasmid containing all three recognition sequences was then created, termed pDSB (Fig. 1a). pDSB was incubated sequentially in High Speed Supernatant (HSS) and NucleoPlasmic Extract (NPE), which promotes replication^26^ and chromatinization of plasmid DNA^27–30^. To monitor the formation and repair of DSBs, reactions were supplemented with radiolabeled nucleotide ([α-^32^P] dATP), which is incorporated into nascent strands during synthesis^26^.

pDSB was replicated in extract for 45 min to allow the formation of open circular (OC) and supercoiled (SC) plasmids (Fig. 1b, lanes 1–5). The reaction was then split and supplemented with buffer or AgeI (designated as +0 min). With AgeI addition, most plasmids were linearized within 10 min, indicating efficient DSB formation (Fig. 1b and b). After 60 min, linear molecules were replaced by high molecular weight (HMW) molecules that represent highly branched intermediates formed by HR between multiple plasmids^31^ (Fig. 1b, d). AgeI addition also induced Chk1 phosphorylation (Fig. 1e), indicating activation of the DNA damage response^32^. Similar results were observed in reactions supplemented with KpnI (Supplementary Fig. 1a–d) or EcoRV (Supplementary Fig. 1e–h), although the efficiency of DSB formation varied for each enzyme.

The intermediates of DSB repair were further analyzed by digesting DNA samples with AvrII and resolving them by 2D agarose gel electrophoresis (Supplementary Fig. 2a). With AgeI addition, we observed the predicted DSB fragments (referred to as “Short” and “Long”), which form a “comet” shape in 2D gels due to nucleolytic resection of DNA ends^33^ (Supplementary Fig. 2b, panel 2). Resection is the first step of HR, which creates a 3’ single-stranded DNA end required for strand invasion. DSB formation also led to an accumulation of branched intermediates that correspond to the HMW HR intermediates in Fig. 1b. In addition, we observed two spots whose migration was consistent with mismatched joining of “Short-Short” or “Long-Long” DSB fragments by NHEJ. These results suggested that both HR and NHEJ pathways were active in our system. Although HR was the dominant mechanism of repair, formation of HR products was sensitive to inhibition of the NHEJ factor DNA-PK (Supplementary Fig. 2b, g), indicating direct competition between both repair pathways.

To identify the mechanisms of end joining, we used amplicon sequencing to analyze DSB junctions (Fig. 1a, “SEQ”) following addition of AgeI, KpnI, or EcoRV. We saw a significant number of insertion products in AgeI, but not KpnI or EcoRV reactions (Fig. 1f). These products correspond to fill-in of 5′ overhangs created by AgeI cleavage (Supplementary Fig. 3). When reactions were supplemented with a DNA-PK inhibitor (DNA-PKi), AgeI insertion products were reduced 15-fold, indicating that they were formed by NHEJ (Fig. 1f). AgeI reactions also formed deletion products of various sizes (Fig. 1g) that were not seen with KpnI or EcoRV (Fig. 1h and i). We hypothesize that KpnI fragments are primed for HR by the presence of 3′ overhangs and that blunt EcoRV fragments are ligated without terminal processing, thereby avoiding deletion products in these reactions. The majority of AgeI deletion products were ≥10 nucleotides in length and contained 2–4 nucleotides of microhomology at the junction (Supplementary Fig. 3), indicative of A-EJ. With the addition of DNA-PKi, AgeI deletions ≥10 nucleotides increased 1.5-fold, compared to buffer controls (Fig. 1g). Thus, inhibition of NHEJ leads to a loss of AgeI insertion products and a concomitant increase in larger A-EJ deletion products. Collectively, these data indicate that the HR, NHEJ, and A-EJ pathways all compete for repair of DSBs and that the composition of DNA ends influences repair pathway choice.

### BET inhibition disrupts A-EJ and HR-mediated repair

**Fig. 2:**
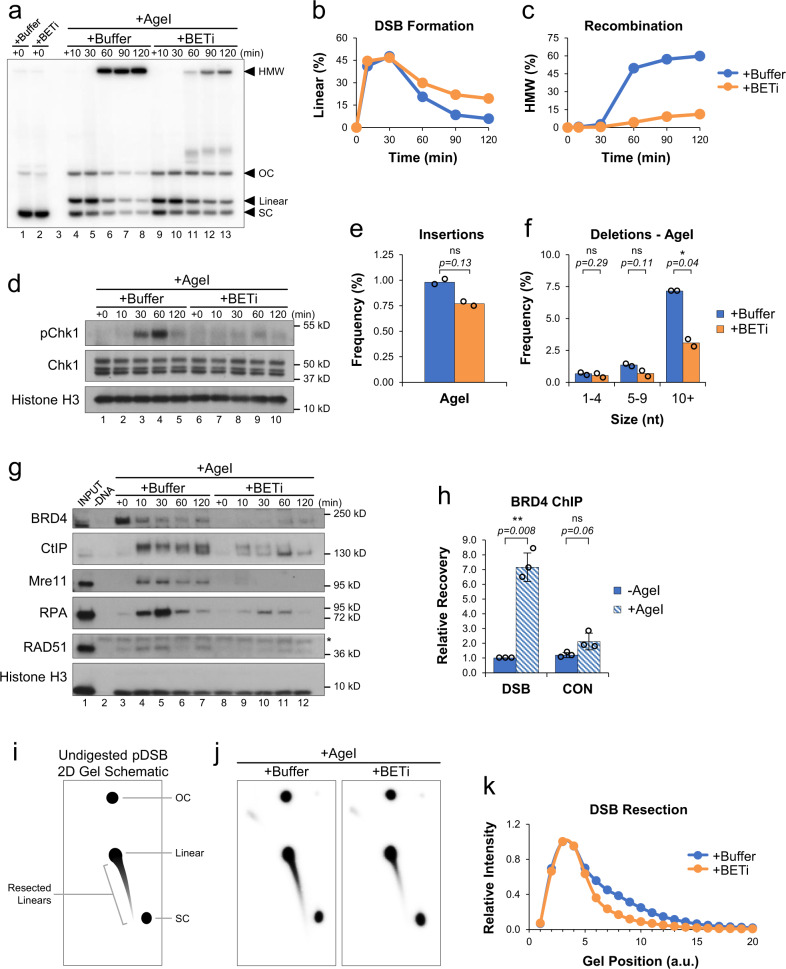
BET proteins promote DNA end resection. **a** pDSB was replicated with α^32^P[dATP] in reactions containing buffer or JQ1 (BETi). After 45 min, reactions were supplemented with AgeI and samples were withdrawn for 1D gel electrophoresis (*n* = 4 independent experiments). **b**, **c** Quantitation of linear (**b**) and HMW (**c**) molecules from (**a**). **d** Protein samples from (**a**) were withdrawn and analyzed by Western blot (*n* = 2 independent experiments). **e**, **f** pDSB was replicated in extract containing buffer or JQ1 (BETi). After 45 min, reactions were supplemented with AgeI. Samples were withdrawn 30 min after enzyme addition and analyzed by amplicon sequencing (*n* = 2 independent experiments). Results are graphed to show the frequency of insertion products (**e**), and the frequency of different deletion products (**f**). **g** pDSB was replicated in extract supplemented with buffer or JQ1 (BETi). After 45 min, AgeI was added and DNA-bound proteins were isolated by plasmid pull-down. Samples were analyzed by Western blot with the indicated antibodies (*n* = 2 independent experiments). Non-specific band (*). **h** pDSB and a control plasmid lacking AgeI sites were replicated in extract. After 45 min, AgeI was added and samples were withdrawn 5 min later for analysis by BRD4 ChIP (*n* = 3 independent experiments). **i** Schematic of undigested 2D gel intermediates. The relative position of open circular (OC), supercoiled (SC), and linear plasmids is indicated. An example of resected linear molecules is also shown. **j** pDSB was replicated with α^32^P[dATP] in reactions containing buffer or BETi. After 45 min, AgeI was added and samples were withdrawn 30 min later for 2D gel electrophoresis (*n* = 2 independent experiments). **k** Quantitation of linear and resected molecules in (**j**). Arbitrary units (a.u.). Error bars represent ± one standard deviation from the mean. Student’s two-tailed *t* test: not significant (ns), *p*-value < 0.05 (*), *p*-value < 0.01 (**).

BET proteins are known to regulate expression of various DNA repair genes^8^. To test whether BET proteins also play a direct role in DSB repair, we supplemented reactions with buffer or the highly selective BET inhibitor, JQ1. JQ1 (BETi) is an acetyl-lysine mimic that blocks interaction of BET proteins with chromatin^34,35^. In the presence of BETi, we saw that linear molecules persisted (Fig. 2a, b) and that accumulation of HMW HR intermediates was severely reduced (Fig. 2a, c). Similar results were also seen with two other BET inhibitors (Supplementary Fig. 4a–f). Displacement of BRD4 from DNA by BET inhibition coincided with loss of HR intermediates (Supplementary Fig. 4g, h), suggesting the two events are linked. DNA damage signaling was also disrupted by BET inhibition (Fig. 2d). Reactions where pDSB was cleaved by KpnI or EcoRV had similar defects in damage signaling and HR (Supplementary Fig. 5a–h). Together, these data indicate that BET proteins play a critical role in promoting HR-mediated repair of DSBs containing various types of DNA ends.

To test whether BET inhibition also influenced DNA repair by NHEJ, we analyzed DSB junctions in AgeI reactions supplemented with buffer or BETi. In the presence of BETi, formation of insertion products was not significantly affected (Fig. 2e). However, deletions ≥10 nucleotides were reduced over 2-fold by BET inhibition (Fig. 2f). These data suggest that BET proteins play a relatively limited role in NHEJ but play a prominent role in promoting A-EJ. Notably, both A-EJ and HR involve nucleolytic resection of DNA ends, highlighting a common mechanism whereby BET proteins could promote DSB repair by both pathways. Importantly, our observations do not rule out a more significant role for BET proteins in NHEJ under different contexts. For example, BET proteins may have an alternative function outside S/G2 phase when HR is suppressed^36,37^. Different forms of cellular stress may also lead to epigenetic changes that influence BET localization or activity^38^.

### Chromatin signaling links transcription and HR activity

Studies have found that HR is elevated in transcriptionally active chromatin^39–41^. We recently showed that NPE can support transcription of plasmid substrates^30^, allowing us to distinguish between the effects of an active transcription complex and the more general consequences of chromatin accessibility associated with transcribed chromatin. To inhibit the transcription complex, reactions were supplemented with the RNAPII inhibitor α-amanitin (RNAPIIi). RNAPII inhibition had no effect on DSB repair with or without BETi (Supplementary Fig. 6a–d), indicating that RNAPII is dispensable for DSB repair and that BET proteins promote HR through a transcription-independent mechanism. We then sought to test whether chromatin signaling associated with transcriptional activity could stimulate DSB repair. Reactions were supplemented with Vorinostat, a pan class I, II, and IV histone deacetylase inhibitor (HDACi) that promotes chromatin decondensation^42,43^. In the presence of HDACi, we saw that resolution of linear molecules and formation of HMW HR intermediates occurred more quickly (Supplementary Fig. 6e–h). Thus, HDAC inhibition accelerates repair of DSBs by HR. These results support observations made in cells^39,41,44^ and further underscore the association between chromatin accessibility and HR activity.

### BET proteins promote DNA end resection

To investigate how BET proteins stimulate repair, we isolated pDSB from extract by plasmid pull-down and visualized DNA-bound proteins by Western blot. In the buffer control reaction, we saw that BRD4 had accumulated on pDSB prior to AgeI addition and that the level of total bound protein was significantly reduced thereafter (Fig. 2g, lanes 3–7), suggesting that its role in repair occurs shortly after DSB formation. Consistent with this interpretation, analysis of protein binding by ChIP (Fig. 1a, “ChIP”) revealed a damage-dependent increase in BRD4 binding near the DSB (Fig. 2h). In the presence of BETi, BRD4 binding was blocked (Fig. 2g, lanes 8-12). BET inhibition also reduced DNA binding of the resection nucleases CtIP and Mre11, the ssDNA binding protein RPA, and the RAD51 recombinase that promotes strand invasion during HR. Together, these results argue that BET proteins promote HR through the recruitment of resection machinery and subsequent loading of RPA and RAD51 onto resected DNA.

To examine the extent of DSB resection, DNA was isolated 30 min after AgeI addition and resolved by 2D agarose gel electrophoresis without AvrII digestion (Fig. 2i). Cleavage by AgeI created a linear spot that formed a comet due to DNA end resection (Fig. 2j). Compared to the buffer control, BET inhibition reduced both the length and intensity of the comet tail (Fig. 2j, k), indicating that BET proteins promote resection of DSB ends. Initial resection of DNA ends involves the coordinated action of CtIP and the MRN complex^45–47^. To confirm that resection occurred through a canonical mechanism, resection was also analyzed in mock- or CtIP-depleted extract (Supplementary Fig. 7a)^48,49^. In the absence of CtIP, resection was completely blocked, preventing resolution of linear molecules and formation of HMW HR intermediates (Supplementary Fig. 7b–f). These data show that CtIP is essential for HR in extract. Thus, BET-mediated recruitment of CtIP is critical to promote efficient resection of DNA ends.

### BET-mediated BRG1 recruitment and histone eviction

**Fig. 3:**
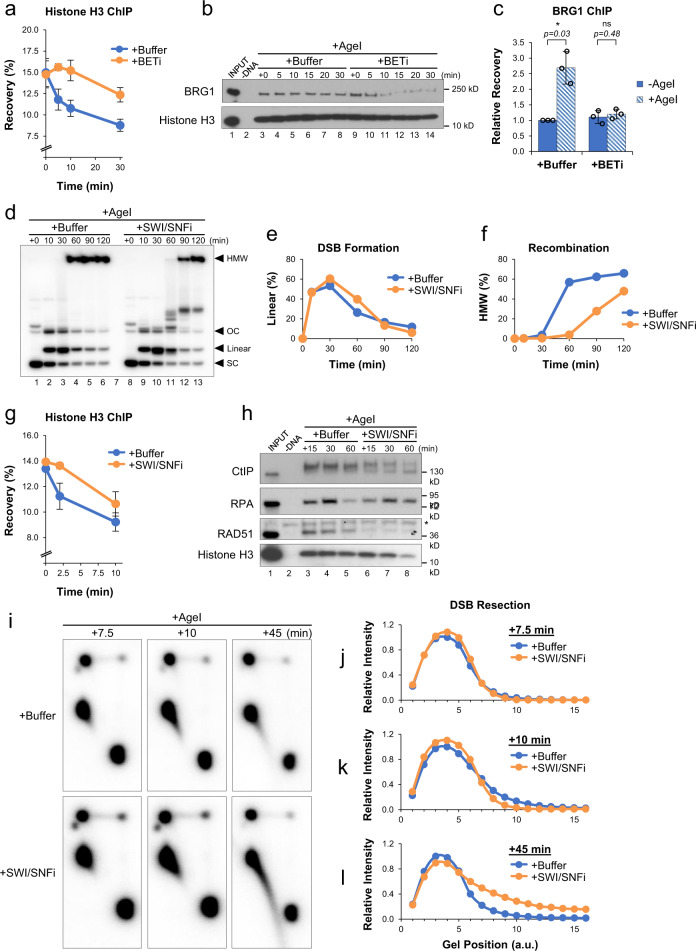
BET-mediated regulation of SWI/SNF. **a** pDSB was replicated in extract supplemented with buffer or JQ1 (BETi). After 45 min, AgeI was added and samples were withdrawn for analysis by histone H3 ChIP (*n* = 2 independent experiments). **b** pDSB was replicated in extract supplemented with buffer or BETi. After 45 min, AgeI was added and DNA-bound proteins were isolated by plasmid pull-down. Samples were analyzed by Western blot with the indicated antibodies (*n* = 2 independent experiments). **c** pDSB was replicated in extract supplemented with buffer or JQ1 (BETi). After 45 min, AgeI was added and samples were withdrawn 5 min later for analysis by BRG1 ChIP (*n* = 3 independent experiments). **d** pDSB was replicated with α^32^P[dATP] in extract containing buffer or the BRG1/BRM ATP Inhibitor-1 (SWI/SNFi). After 45 min, reactions were supplemented with AgeI and samples were withdrawn for 1D gel electrophoresis (*n* = 2 independent experiments). **e**, **f** Quantitation of linear (**e**) and HMW (**f**) molecules from (**d**). **g** pDSB was replicated in extract supplemented with buffer or SWI/SNFi. After 45 min, AgeI was added and samples were withdrawn for analysis by histone H3 ChIP (*n* = 2 independent experiments). **h** pDSB was replicated in extract containing buffer or SWI/SNFi. After 45 min, reactions were supplemented with AgeI and DNA-bound proteins were isolated by plasmid pull-down. Samples were analyzed by Western blot with the indicated antibodies (*n* = 2 independent experiments). Non-specific band (*). **i** pDSB was replicated with α^32^P[dATP] in extract containing buffer or SWI/SNFi. After 45 min, reactions were supplemented with AgeI and samples were withdrawn for 2D gel electrophoresis (*n* = 3 independent experiments). **j**–**l** Quantitation of linear and resected molecules in (**i**) at 7.5 (**j**), 10 (**k**), and 45 (**l**) minutes after AgeI addition. Arbitrary units (a.u.). Error bars represent ± one standard deviation from the mean. Student’s two-tailed *t* test: not significant (ns), *p*-value < 0.05 (*).

Chromatin remodeling is an essential component of the DNA damage response that allows repair machinery to access DNA^50^. To investigate whether BET proteins regulate resection by altering the chromatin landscape, we analyzed histone H3 binding by ChIP. Normally, AgeI addition leads to a rapid decrease in histone binding near the DSB (Fig. 3a, blue trace). In contrast, histone eviction was severely delayed (≥30 min) by BET inhibition (Fig. 3a, orange trace), indicating that BET proteins play a role in remodeling chromatin at DSBs. Brahma-Related Gene 1 (BRG1) is an ATPase subunit of the SWI/SNF chromatin remodeling complex, and has been linked to both DNA repair^51–53^ and BET proteins^54,55^. To test whether BET proteins regulate binding of BRG1 during DSB repair, DNA-bound proteins were analyzed by plasmid pull-down. In the buffer control, BRG1 remained bound to pDSB throughout the reaction (Fig. 3b, lanes 3–8). However, in the presence of BETi, binding of BRG1 was reduced after AgeI addition (Fig. 3b, lanes 9–14), arguing that BET proteins are important to maintain association of SWI/SNF with damaged chromatin. Indeed, when we analyzed recruitment of BRG1 to the DSB region by ChIP, we saw that BRG1 binding increased in response to damage, and that the increase was blocked by BET inhibition (Fig. 3c).

### SWI/SNF inhibition delays resection and blocks RAD51 loading

SWI/SNF has been proposed to function at multiple steps of DSB repair, including stimulating DNA end resection^51,56^ and promoting the exchange of RPA for RAD51 after resection^57^. To test whether the repair defects associated with BET inhibition could be attributed to the loss of SWI/SNF activity, we supplemented extract with buffer or an inhibitor of the SWI/SNF ATPase subunits BRG1/BRM (SWI/SNFi)^58^. Compared to the buffer control, SWI/SNF inhibition severely reduced accumulation of HMW HR intermediates (Fig. 3d, f). We also saw an interesting effect of SWI/SNFi on linear molecules, whose resolution was initially delayed, but then surpassed that of the buffer control (Fig. 3d, e). These results argue that BET proteins promote repair, in part, through SWI/SNF recruitment, but that SWI/SNF’s role in DSB repair is mechanistically distinct from that of BET proteins.

To further investigate the mechanism of SWI/SNF-mediated repair, we analyzed histone H3 binding by ChIP. Compared to a buffer control, reactions supplemented with SWI/SNFi showed a modest delay (~10 min) in histone H3 eviction (Fig. 3g). We also analyzed DNA-bound proteins and found that SWI/SNF inhibition reduced the accumulation of CtIP (Fig. 3h), as seen with BET inhibition (Fig. 2g). However, in the presence of SWI/SNFi, RPA accumulation was not blocked, but simply delayed (Fig. 3h). 2D gels showed that resection was reduced at 10 min (Fig. 3i, k), but increased dramatically by 45 min (Fig. 3i, l). Thus, SWI/SNF inhibition delays, but does not block, resection of DNA ends. BRD4 is reported to have nucleosome eviction capability^59^, which may partially compensate for histone eviction by SWI/SNF. However, despite the high levels of resection and RPA seen at later times, RAD51 binding was completely blocked by SWI/SNF inhibition (Fig. 3h). These results argue that SWI/SNF’s most important function in DSB repair is loading RAD51^57^, which is required to promote HR and avoid excessive resection.

### The role of BRD4 in DSB repair

**Fig. 4:**
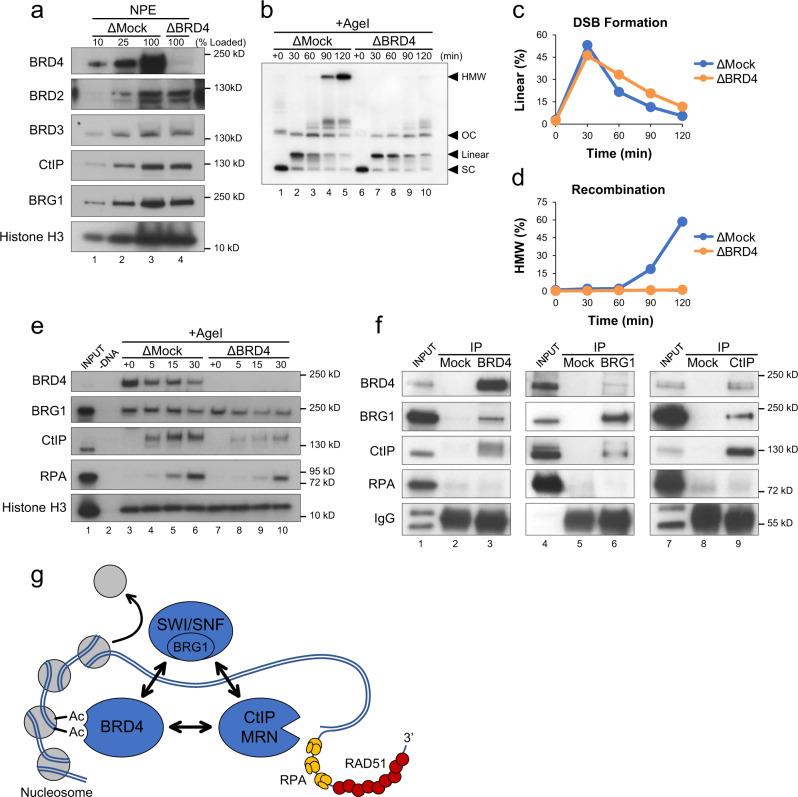
BRD4 promotes resection and homology-directed repair. **a** Mock-depleted (ΔMock) or BRD4-depleted (ΔBRD4) NPE was analyzed by Western blot using the indicated antibodies (*n* = 2 independent experiments). **b** pDSB was replicated with α^32^P[dATP] in mock- or BRD4-depleted extracts. After 60 min, reactions were supplemented with AgeI and samples were isolated at the indicated time points for 1D gel electrophoresis (*n* = 2 independent experiments). **c**, **d** Quantitation of linear (**c**) and HMW (**d**) molecules from (**b**). **e** pDSB was replicated in mock- or BRD4-depleted extract. After 60 min, reactions were supplemented with AgeI and DNA-bound proteins were isolated by plasmid pull-down. Samples were analyzed by Western blot with the indicated antibodies (*n* = 2 independent experiments). **f** A mixture of HSS and NPE was immunoprecipitated with beads conjugated to mock, BRD4, BRG1, or CtIP antibodies. Bead-bound proteins were analyzed by Western blot with the indicated antibodies (*n* = 3 independent experiments). **g** Schematic showing interactions between BRD4-BRG1-CtIP and their role in coordinating DSB repair through nucleosome eviction, DNA end resection, and homologous recombination. Acetylation (Ac); Mre11-Rad50-Nbs1 (MRN).

Most studies of BET function rely on inhibitors that target multiple BET proteins^34,60–62^. To investigate the specific contributions of BRD2, BRD3, and BRD4 in DSB repair, we raised antibodies against each *Xenopus laevis* protein. Profiles of protein and mRNA levels during *Xenopus* development indicate that eggs are highly enriched for BRD4 compared to BRD2 and BRD3^63–65^. Although BRD2 and BRD3 antibodies supported little or no immunoprecipitation (Supplementary Fig. 8a, b), BRD4 antibodies readily depleted BRD4 without co-depletion of BRD2 or BRD3 (Fig. 4a), allowing us to investigate BRD4’s specific contribution to DSB repair. We found that depletion of BRD4 led to similar defects in repair as BET inhibition. In the absence of BRD4, resolution of linear molecules and formation of HMW HR intermediates were both delayed (Fig. 4b–d). Loss of BRD4 also led to a decrease in DNA binding of BRG1, CtIP, and RPA (Fig. 4e). Taken together, these results specifically implicate BRD4 in promoting the resection and homology-directed repair of DSBs.

### Multiple interactions connect BRD4-BRG1-CtIP

BRD4 has been reported to interact with BRG1^66^. To further explore the relationship between BRD4 and downstream effectors, we performed reciprocal co-immunoprecipitations of BRD4, BRG1, and CtIP. We saw that all three proteins were recovered by each immunoprecipitation (Fig. 4f). Notably, the recovery of associated proteins was modest, suggesting that they do not typically form a stable complex. Indeed, Fig. 4a showed that BRD4 depletion did not co-deplete either CtIP or BRG1. We also saw that BRD4’s interaction with BRG1 and CtIP was not dependent on its bromodomains (Supplementary Fig. 8c). We hypothesize that each protein may be recruited to chromatin individually, where interactions then facilitate coordinated processing and homology-directed repair of DSBs (Fig. 4g).

## Discussion

Here, we identify a direct molecular function for BRD4 in DSB repair. We show that BRD4 orchestrates repair pathway choice through a coordinated series of events that includes (1) recruitment of SWI/SNF chromatin remodeling complexes, (2) displacement of histones from the DSB, and (3) DNA end resection to promote RAD51 loading and HR. Although our data specifically implicate BRD4, we do not rule out the possibility that other BET proteins could compensate for BRD4 in some contexts. These results expand our understanding of BET proteins, establishing them as multifunctional regulators of chromatin binding that control how different cellular processes interact with DNA. Both of BRD4’s gene expression-dependent and –independent functions are critical for efficient DNA repair, suggesting that BRD4 is a central mediator through which damage signaling can be controlled. Although the BET protein family has an established role in cancer as regulators of oncogenes like *C-MYC*, *CCND1*, *KRAS*, *BCL2*, and *BRAF*^10–13^, the use of BET inhibitors as single agents has had limited success in the clinic^19^. Many preclinical studies suggest that BET inhibitors may be more effective in combination with existing therapeutics^19^. Intriguingly, BET inhibition has been found to synergize with multiple agents that target DNA damage signaling and repair, including HDAC inhibitors^67–69^, PARP inhibitors^16,17,70,71^, topoisomerase inhibitors^72,73^, and genotoxic agents like cisplatin^74,75^. Our findings highlight the critical role that BET proteins play in regulating multiple aspects of genome maintenance and underscore their potential as pharmacological targets for developing new and more effective cancer therapies.

## Methods

### Double-strand break reactions

To create pDSB, an EcoRV site was cloned into pCMV-GFP (Addgene ##11153) using the following primer pairs (QuikChange, Agilent): CGCTACCGGACTCAGATATCGAGCTCAAGCTT and AAGCTTGAGCTCGATATCTGAGTCCGGTAGCG. Replication reactions were performed as described previously^76^. Briefly, 2.5–5 ng/μL pDSB was incubated in HSS supplemented with ATP Regeneration Mix (ARM; 6.5 mM phosphocreatine, 0.65 mM ATP, and 1.6 μg/mL creatine phosphokinase) and 10 μM nocodazole for 20 min at 21 °C to form pre-Replication Complexes. To trigger replication, NPE supplemented with ARM and 3.5 mM DTT was added at a 2:1 ratio and reactions were incubated at 21 °C for an additional 45-60 min. To induce DSBs, reactions were supplemented with AgeI (0.25 U/μL), KpnI (.091 U/μL), or EcoRV (20 U/μL) from New England Biolabs. Enzyme storage buffer (10 mM Tris-HCl, 250 mM NaCl, 1 mM DTT, 0.1 mM EDTA, 200 μg/mL, 50% glycerol, 0.15% TritonX-100 pH 7.4) was used as a buffer control. Where indicated, reactions were also supplemented with 0.3 μCi/μL α^32^P[dATP], 18 μM BRC peptides^31^, 87 μM NU7441 (Selleckchem), 300 μM JQ1 (Sigma), 1,000 μM I-BET 762 (SelleckChem), 500 μM MS417 (MedChemExpress), 1,000 μM BRG1/BRM ATP Inhibitor-1 (MedChemExpress), 20 ng/μL α-amanitin (Sigma), or 100 μM Vorinostat (Cayman Chemical). All reactions were performed at least twice and representative data are shown. Student t-tests were performed using Microsoft Excel (v14.7.7).

### Antibodies, immunodepletion, and immunoprecipitation

Commercial antibodies were used to detect phosphorylated Chk1 (Cell Signaling, #2341, 1:1000 dilution), Chk1 (Bethyl Laboratories, A300-298A, 1:4000 dilution), histone H3 (Thermo Fisher, PA5-16183, 1:4000 dilution), and BRG1 (Bethyl Laboratories, A300-813A, 1:4000 dilution). *Xenopus laevis* BRD2, BRD3, and BRD4 antibodies were produced by New England Peptide (NEP) using the following antigen sequences: BRD2-KPHDKAESAHQVSVT, BRD3-EPRRERYKGATQAS, and BRD4-NFQSELMEIFEQNLFS (1:4000 dilution). *Xenopus laevis* Mre11 and CtIP antibodies were generously provided by the laboratories of Jean Gautier and Richard Baer from Columbia University^48,77^ (1:4000 dilution). *Xenopus laevis* RAD51 and RPA antibodies were developed previously^31,78^ (1:4000 dilution).

Immunodepletions were performed as described previously^30,35^. Briefly, to immunodeplete BRD4 or CtIP, 16 μL of serum or 200 μg of purified IgGs was conjugated to 4 μL of Protein A Sepharose Fast Flow beads (VWR) and incubated with 10 μL of NPE at 4 °C for 1 h over two rounds. For mock-depleted controls, an identical immunodepletion was performed in parallel with pre-immune serum. Depleted extracts were isolated from beads by Nytex filtration and used immediately for experiments. HSS was depleted as described above for one round, and the resulting CtIP- or BRD4-depleted HSS was used for reactions with both mock- and protein-depleted NPE.

For immunoprecipitations, 5 μL of the indicated antibody was conjugated to 5 μL of Protein A Sepharose Fast Flow beads. A mixture of HSS and NPE was diluted 8-fold in IP Buffer (10 mM HEPES-KOH pH 7.7, 2.5 mM MgCl_2_, 50 mM KCl, 250 mM sucrose, and 0.02% Tween-20) and incubated with beads at 4 °C for 90 min. Beads were then washed 4 times with IP Buffer and resuspended in 2x SDS PAGE Buffer (100 mM Tris-HCl pH 7.5, 20% glycerol, 4% SDS, 200 mM β-mercaptoethanol, and 0.2% bromophenol blue). Bead-bound proteins were then resolved by SDS PAGE and visualized by Western blot with the indicated antibodies.

### Agarose Gel Electrophoresis

For 1D agarose gel electrophoresis, 1 μL of reaction was diluted 6-fold in Replication Stop Dye (3.6% SDS, 18 mM EDTA, 90 mM Tris-HCl pH 8, 90 mg/mL Ficoll, and 3.6 mg/mL Bromophenol Blue), incubated with 20 mg proteinase K at 21 °C for 16 h, and then resolved by 0.8% agarose gel electrophoresis. For 2D agarose gel electrophoresis, 1.5 μL of reaction was diluted 10-fold in Stop Solution (50 mM Tris-HCl pH 7.5, 25 mM EDTA, and 0.5% SDS), incubated with 4 mg RNase for 30 min at 37 °C, and then incubated with 30 mg proteinase K at 21 °C for 16 h. DNA was isolated by phenol/chloroform extraction, followed by ethanol precipitation. Undigested or AvrII-digested (New England Biolabs) DNA intermediates were then resolved by 0.4% agarose gel electrophoresis at 1 V/cm for 14 h. The 1D gel was then stained with 0.3 μg/mL ethidium bromide and individual lanes were cut out. For the 2nd dimension, 1% agarose containing 0.3 μg/mL ethidium bromide was cast around the 1D slices and the gel was run in buffer containing 0.3 μg/mL ethidium bromide at 4 V/cm for 15 h at 4 °C. After electrophoresis, agarose gels were dried and visualized by autoradiography. Linear and HMW molecules were quantified from the gel images shown and graphed as a percent of the total signal prior to DSB formation (+0 min). Resection of linear fragments was quantified in sections using boxes that start above the linear spot and move along the length of the comet tail. The intensity at each position was graphed relative to the +Buffer or ΔMock control.

### DSB sequencing analysis

DNA was purified from reactions 30 min after restriction enzyme addition. Intact DSB junctions were amplified by qPCR using the following primers: GGTAGGCGTGTACGGTGGGA and CGTAGGTGGCATCGCCCTCG, which span the span the restriction enzyme sites (Fig. 1a). qPCR reactions were stopped in the linear range of amplification, thus preventing over-amplification of products based on limiting reagents. The resulting 270-nucleotide PCR fragments were resolved by agarose gel electrophoresis and isolated by gel extraction (Qiagen). Amplicon sequencing was then performed by Genewiz and produced ~50,000 reads per sample. Variant detection analysis was performed for SNPs and INDELs up to 20 bp in length. The mutation frequency represents the percentage of reads identified with each sequence out of the total number of plasmids present in each reaction.

### RNA purification and quantitation

RNA was isolated from extract using the EZNA RNA Purification kit (Omega Bio-tek). cDNA was produced using QuantiTect Reverse Transcription kit (Qiagen) and then analyzed by qPCR. RNA recovery was normalized to endogenous 18S rRNA present in extract. The following primer pairs were used:

pDSB: CAAGATCCGCCACAACATCGAGG and CACGAACTCCAGCAGGACCATG

18S rRNA: GACCGGCGCAAGACGAACCA and TGCTCGGCGGGTCATGGGAA

### Plasmid Pull-Down

Plasmids were isolated from extract as described previously^76^. Briefly, reaction samples were added to LacI-coupled magnetic beads (Dynabeads M-280; Invitrogen) suspended in LacI Pull-Down Buffer (10 mM HEPES pH 7.7, 50 mM KCl, 2.5 mM MgCl_2_, 250 mM sucrose, 0.25 mg/mL BSA, and 0.02% Tween 20). Samples were rotated at 4 °C for 20 min, washed three times with LacI Wash Buffer (10 mM HEPES pH 7.7, 50 mM KCl, 2.5 mM MgCl_2_, 0.25 mg/mL BSA, and 0.02% Tween 20), and resuspended in 2x SDS PAGE Buffer. DNA-bound proteins were then resolved by SDS PAGE, and visualized by Western blot with the indicated antibodies.

### Chromatin Immunoprecipitation (ChIP)

ChIP was performed as described previously^79^. Briefly, reaction samples were crosslinked in Egg Lysis Buffer (ELB: 10 mM HEPES-KOH pH 7.7, 2.5 mM MgCl_2_, 50 mM KCl, and 250 mM sucrose) containing 1% formaldehyde. Crosslinking was stopped with the addition of 125 mM glycine and excess formaldehyde was removed with a Micro Bio-Spin 6 chromatography column (Bio-Rad). Samples were sonicated (Diagenode Bioruptor UCD-600 TS) and immunoprecipitated with the indicated antibodies. Crosslinks were then reversed and the resulting DNA was isolated by phenol/chloroform extraction and ethanol precipitation. Total (INPUT) and recovered DNA were analyzed by qPCR. The following primers were used to amplify sequence adjacent to the AgeI site on pDSB (DSB) or an undamaged control plasmid^31^ (CON):

DSB: GGTAGGCGTGTACGGTGGGA and AAGCTTGAGCTCGATATCTGAGTCCGGTAGCG

CON: AGCCAGATTTTTCCTCCTCTC and CATGCATTGGTTCTGCACTT

### Reporting summary

Further information on research design is available in the Nature Research Reporting Summary linked to this article.

## Supplementary information

Supplementary Information

Reporting Summary

## Data Availability

The sequencing data generated in this study have been deposited to the the Harvard Dataverse Repository, 10.7910/DVN/CEFLQ8. Source data are provided with this paper.

## Source data

Source Data
