# Supplementary material for: BRD4 promotes resection and homology-directed repair of DNA double-strand breaks
Source: Nat Commun. 2022 May 31;13:3016. doi: 10.1038/s41467-022-30787-6 (PMC9156784; doi:10.1038/s41467-022-30787-6)
Supplement: Supplementary file 1 — Supplementary Information [file 41467_2022_30787_MOESM1_ESM.pdf]

Supplemental Information:

BRD4 promotes resection and homology-directed repair of DNA double-strand breaks

John K. Barrows[1], Baicheng Lin[1], Colleen E. Quaas[1], George Fullbright[1], Elizabeth N. Wallace[1], and David T. Long[1]*

[1]Department of Biochemistry and Molecular Biology,

Medical University of South Carolina, Charleston, SC 29425, USA.

*To whom correspondence should be addressed. E-mail: longdt@musc.edu

Contents:

Figures S1-S8

Supplemental References

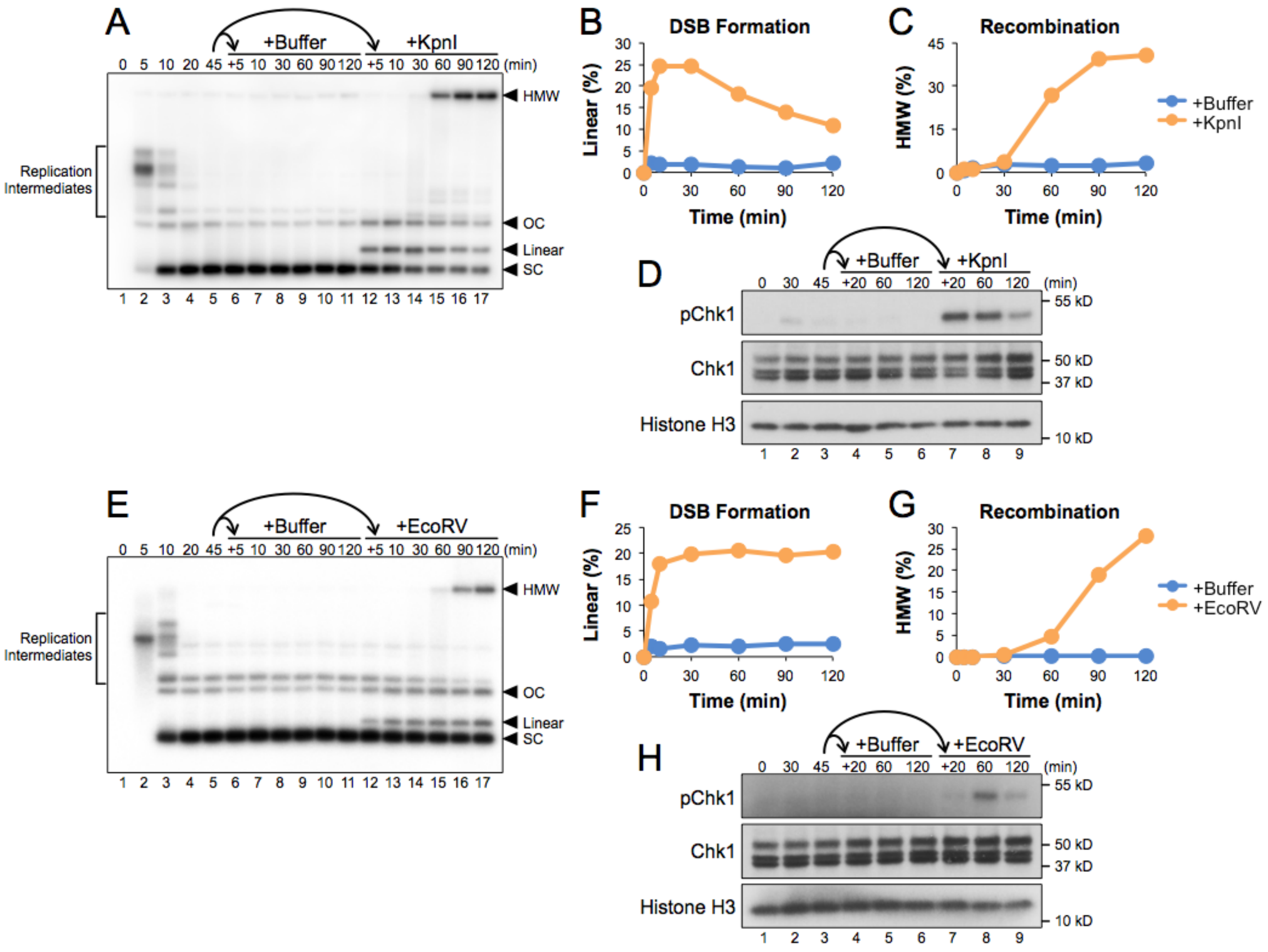

Figure S1. Repair of KpnI- and EcoRV-induced DSBs, Related to Figure 1. (**A**) pDSB was replicated in the presence of α[32]P[dATP] for 45 minutes. The reaction was then split and supplemented with buffer or KpnI. Samples were withdrawn at the indicated time points, resolved by 1D gel electrophoresis, and visualized by autoradiography (n=2 independent experiments). (**B-C**) Quantitation of linear *(B)* and HMW *(C)* molecules from *(A).* (**D**) Protein samples from *(A)* were withdrawn at the indicated time points and analyzed by Western blot with the indicated antibodies (n=2 independent experiments). (**E**) pDSB was replicated as in *(A)* and reactions were supplemented with buffer or EcoRV. Samples were withdrawn at the indicated time points, resolved by 1D gel electrophoresis, and visualized by autoradiography (n=2 independent experiments). (**F-G**) Quantitation of linear *(F)* and HMW *(G)* molecules from *(E).* (**H**) Protein samples from *(E)* were analyzed as in *(D)* (n=2 independent experiments).

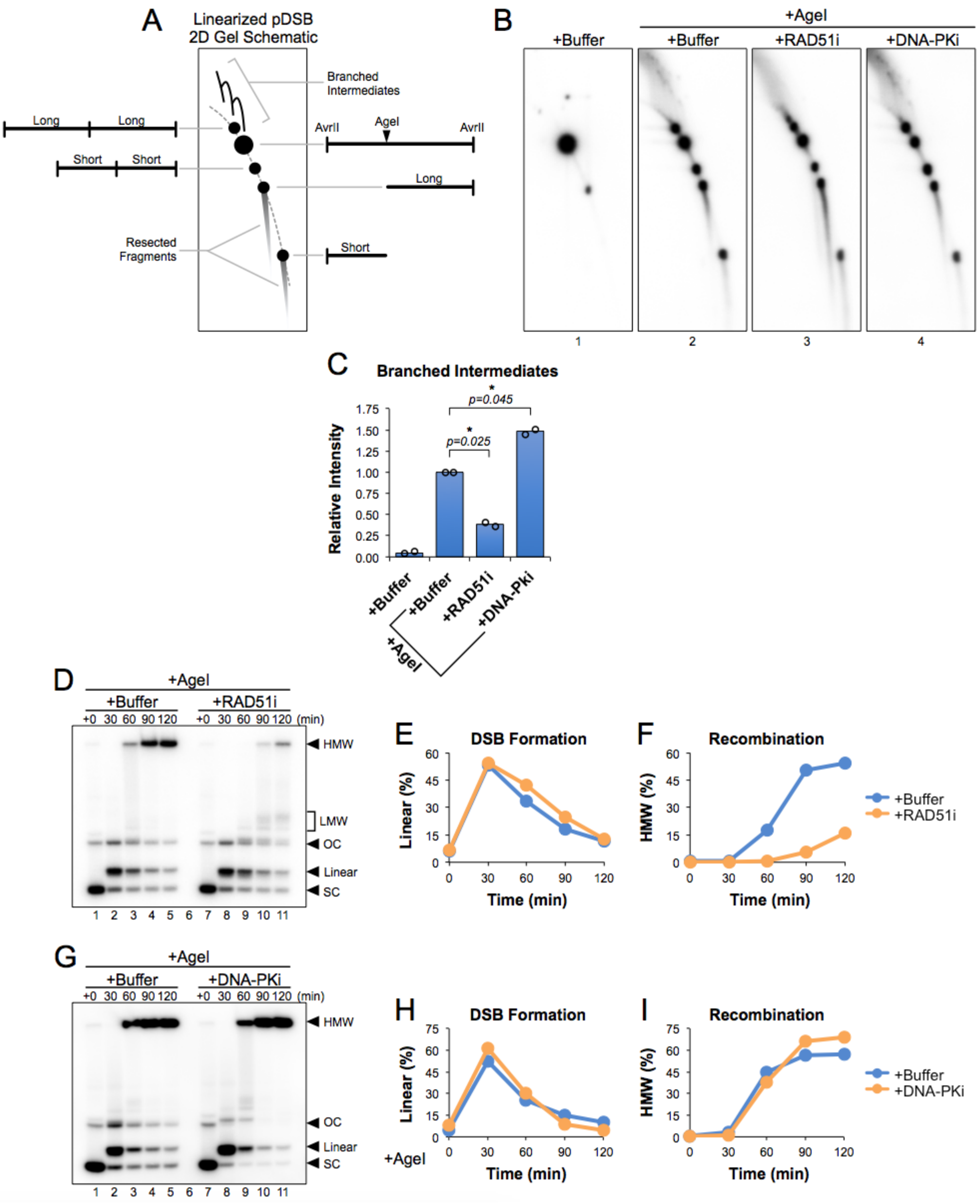
A
Linearized pDSB
2D Gel Schematic
Branched Intermediates
Long
Long
AvrII
AgeI
AvrII
Short
Short
Long
Resected Fragments
Short
B
+AgeI
+Buffer
+Buffer
+RAD51i
+DNA-PKi
1
2
3
4
C
Branched Intermediates
Relative Intensity
p=0.045
p=0.025
+Buffer
+Buffer
+RAD51i
+DNA-PKi
+AgeI
D
+AgeI
+Buffer
+RAD51i
+0 30 60 90 120
+0 30 60 90 120 (min)
HMW
LMW
OC
Linear
SC
1 2 3 4 5 6 7 8 9 10 11
E
DSB Formation
Linear (%)
Time (min)
F
Recombination
HMW (%)
Time (min)
+Buffer
+RAD51i
G
+AgeI
+Buffer
+DNA-PKi
+0 30 60 90 120
+0 30 60 90 120 (min)
HMW
OC
Linear
SC
1 2 3 4 5 6 7 8 9 10 11
H
DSB Formation
Linear (%)
+AgeI
Time (min)
I
Recombination
HMW (%)
Time (min)
+Buffer
+DNA-PKi

Figure S2. Competition between HR and NHEJ in *Xenopus* egg extract, Related to Figure 1. (**A**) Schematic of AvrII-digested 2D gel intermediates. “Short” and “Long” indicate the DSB fragments produced by AgeI cleavage after digestion with AvrII. (**B**) pDSB was replicated in the presence of α^{32}P[dATP]. Reactions were supplemented with buffer, BRC peptides derived from BRCA2 that inhibit RAD51 filament formation and strand invasion (RAD51i)[1-5], or the highly selective DNA-PK inhibitor NU7441 (DNA-PKi). After 45 minutes, reactions were further supplemented with buffer or AgeI. Samples were withdrawn 90 minutes after AgeI addition. DNA was then purified, digested with AvrII, and analyzed by 2D gel electrophoresis (n=2 independent experiments). In the presence of RAD51i peptides (panel 3), the accumulation of branched intermediates was severely reduced compared to the +AgeI buffer control (panel 2). In the presence of DNA-PKi (panel 4), there was a modest increase in the accumulation of branched HR intermediates compared to the +AgeI buffer control (panel 2). (**C**) Quantitation of branched intermediates from experiments described in *(B)* (n=2 independent experiments). (**D**) pDSB was replicated with α^{32}P[dATP] in reactions containing buffer or BRC peptides (RAD51i). After 45 minutes, reactions were supplemented with AgeI and samples were withdrawn at the indicated time points for 1D gel electrophoresis (n=2 independent experiments). (**E-F**) Quantitation of linear *(E)* and HMW *(F)* molecules from *(D)*. In the presence of RAD51i peptides, the decrease of linear molecules was slightly delayed but formation of the corresponding HMW molecules was blocked, arguing that many DSB fragments were degraded due to excessive resection. RAD51 inhibition also led to a small accumulation of low molecular weight (LMW) molecules, which are consistent with limited HR activity involving only two plasmids[6]. These results confirm that repair of AgeI-induced DSBs involves formation of RAD51-dependent branched intermediates, thereby identifying them as *bona fide* HR products. (**G**) pDSB was replicated with α^{32}P[dATP] in reactions containing buffer or NU7441 (DNA-PKi). After 45 minutes, reactions were supplemented with AgeI and samples were withdrawn at the indicated time points for 1D gel electrophoresis (n=2 independent experiments). (**H-I**) Quantitation of linear *(H)* and HMW *(I)* molecules from *(G)*. Addition of DNA-PKi led to a small decrease in linear molecules at later time points and a corresponding increase in HMW molecules, consistent with elevated levels of HR. We also saw that DNA-PK inhibition led to a modest increase in linear molecules 30 minutes after AgeI addition and reduced accumulation of OC and SC bands thereafter, suggesting that some fraction of DSBs are quickly repaired by NHEJ in buffer-treated reactions. Student two-tailed t-test: $p\text{-value} < 0.05$ (*).

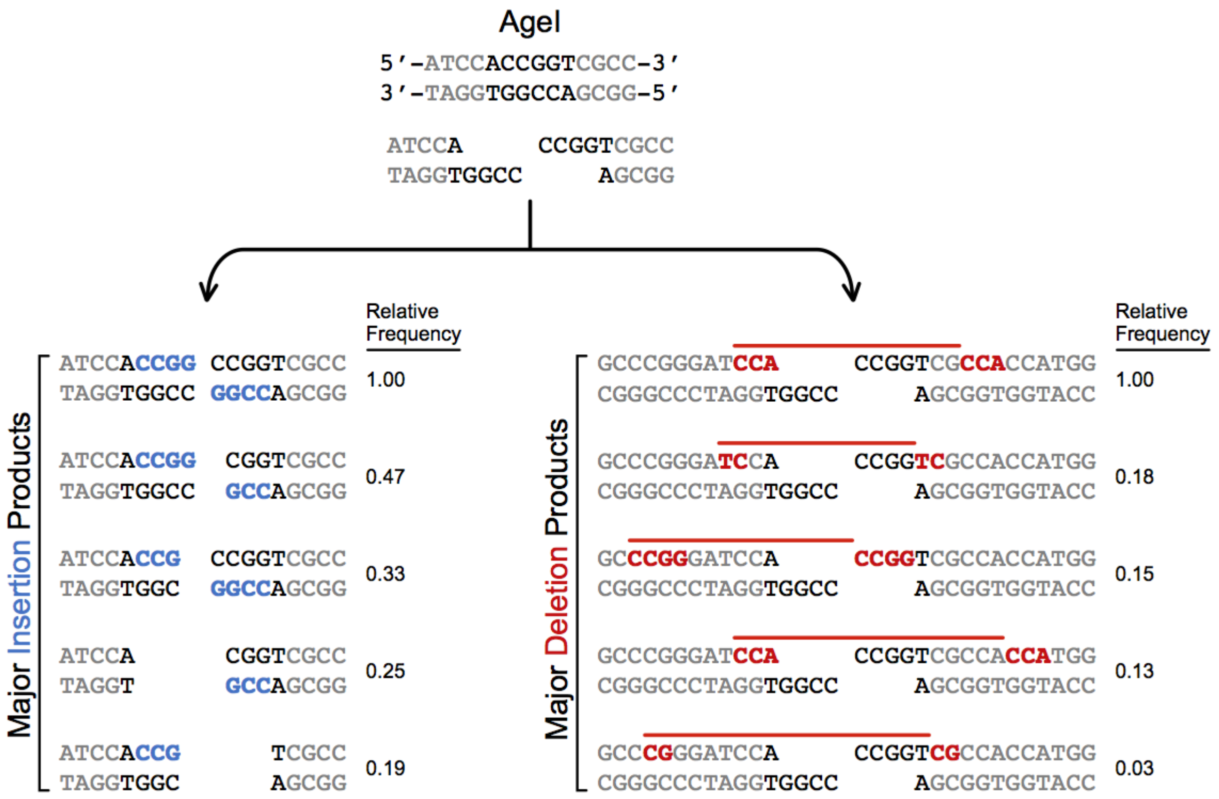

Figure S3. Major insertion and deletion products formed by AgeI-induced DSBs, Related to Figure 1. Schematic showing AgeI cleavage and the major insertion and deletion products revealed by amplicon sequencing. The relative frequency of each product is indicated. Inserted nucleotides are shown in blue. Deleted sequences are denoted by a red line. Nucleotides with microhomology found at the DSB junction are shown in red. Note that sequencing information does not indicate which nucleotides within the microhomology region were removed.

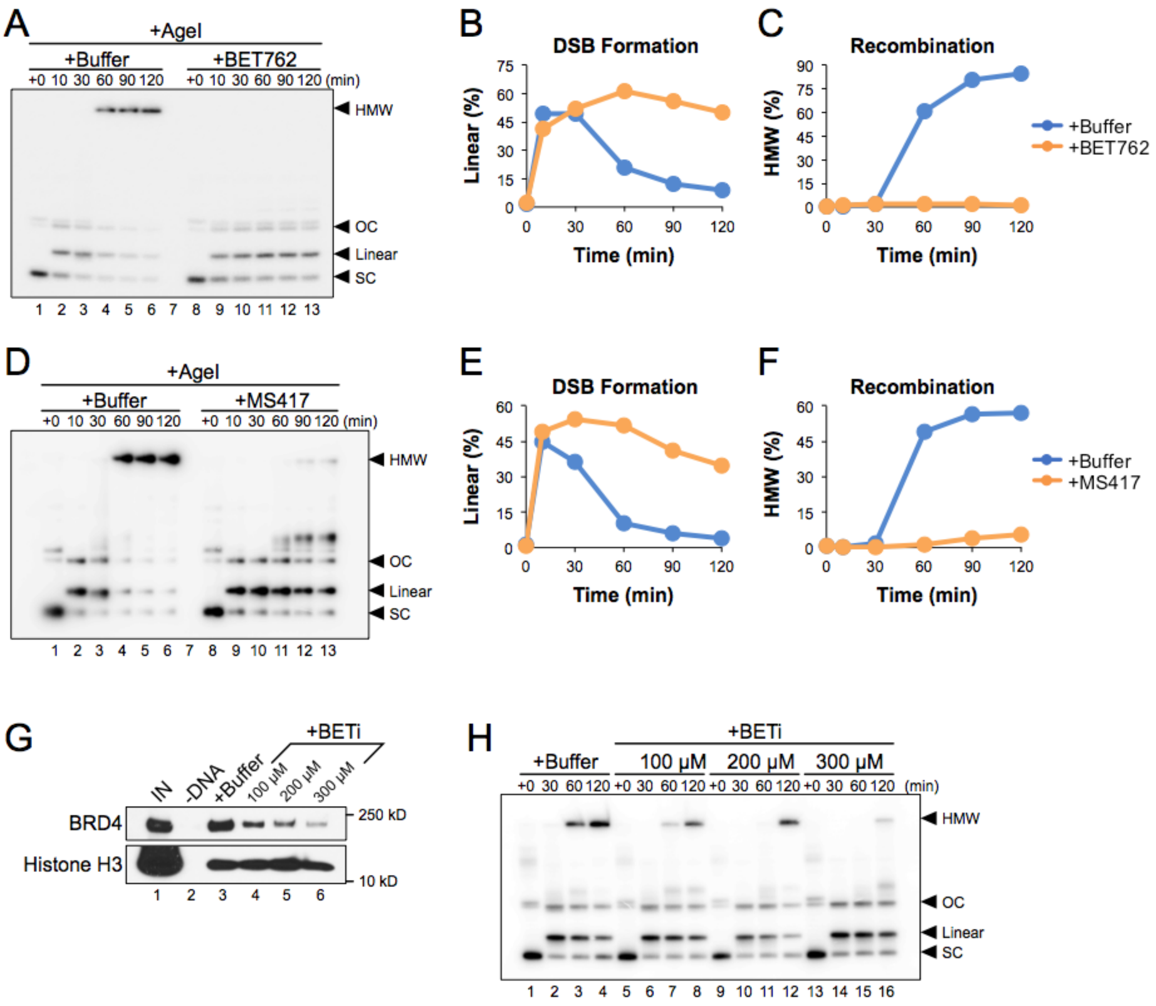

Figure S4. Validation of BET inhibition, Related to Figure 2. (**A**) pDSB was replicated with α^{32}P[dATP] in reactions containing buffer or the BET inhibitor BET762. After 45 minutes, reactions were supplemented with AgeI and samples were withdrawn for 1D gel electrophoresis (n=2 independent experiments). (**B-C**) Quantitation of linear *(B)* and HMW *(C)* molecules from *(A)*. (**D**) pDSB was replicated with α^{32}P[dATP] in reactions containing buffer or the BET inhibitor MS417. After 45 minutes, reactions were supplemented with AgeI and samples were withdrawn for 1D gel electrophoresis (n=2 independent experiments). (**E-F**) Quantitation of linear *(E)* and HMW *(F)* molecules from *(D)*. (**G**) pDSB was replicated in extract supplemented with buffer or the indicated amount of JQ1 (BETi). After 45 minutes, DNA-bound proteins were isolated by plasmid pull-down and analyzed by Western blot with the indicated antibodies (n=2 independent experiments). (**H**) pDSB was replicated with α^{32}P[dATP] in reactions containing buffer or the indicated amount of JQ1 (BETi). After 45 minutes, reactions were supplemented with AgeI and samples were withdrawn for 1D gel electrophoresis (n=2 independent experiments).

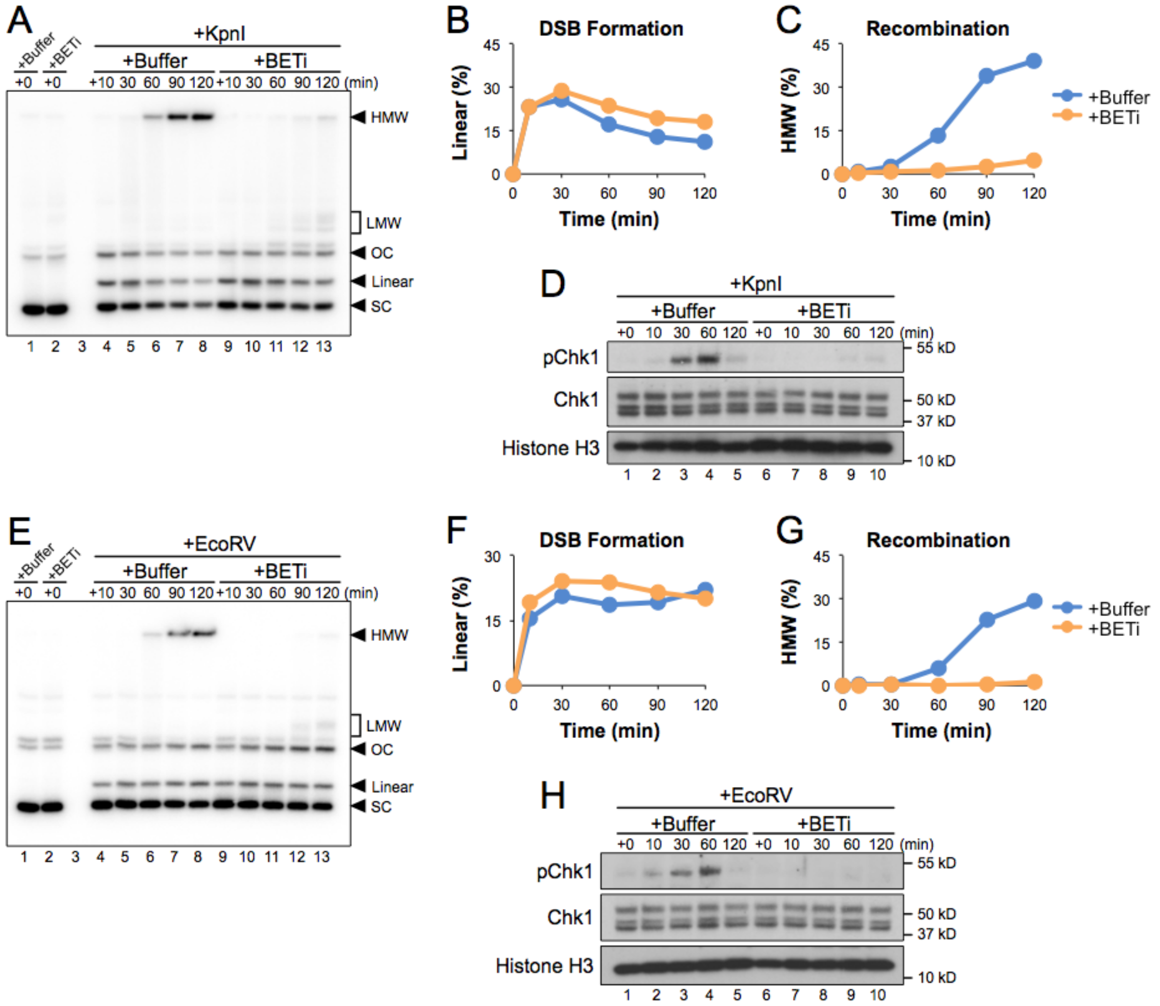

Figure S5. BET inhibition disrupts HR regardless of overhang type, Related to Figure 2. (**A**) pDSB was replicated with α^{32}P[dATP] in reactions containing buffer or JQ1 (BETi). After 45 minutes, reactions were supplemented with KpnI and samples were withdrawn at the indicated time points for 1D gel electrophoresis (n=2 independent experiments). (**B-C**) Quantitation of linear *(B)* and HMW *(C)* molecules from *(A)*. (**D**) Protein samples from *(A)* were withdrawn at the indicated time points and analyzed by Western blot with the indicated antibodies (n=2 independent experiments). (**E**) pDSB was replicated as in *(A)* and reactions were supplemented with buffer or EcoRV. Samples were withdrawn at the indicated time points and analyzed by 1D gel electrophoresis (n=2 independent experiments). (**F-G**) Quantitation of linear *(F)* and HMW *(G)* molecules from *(E)*. (**H**) Protein samples from *(E)* were analyzed as in *(D)* (n=2 independent experiments).

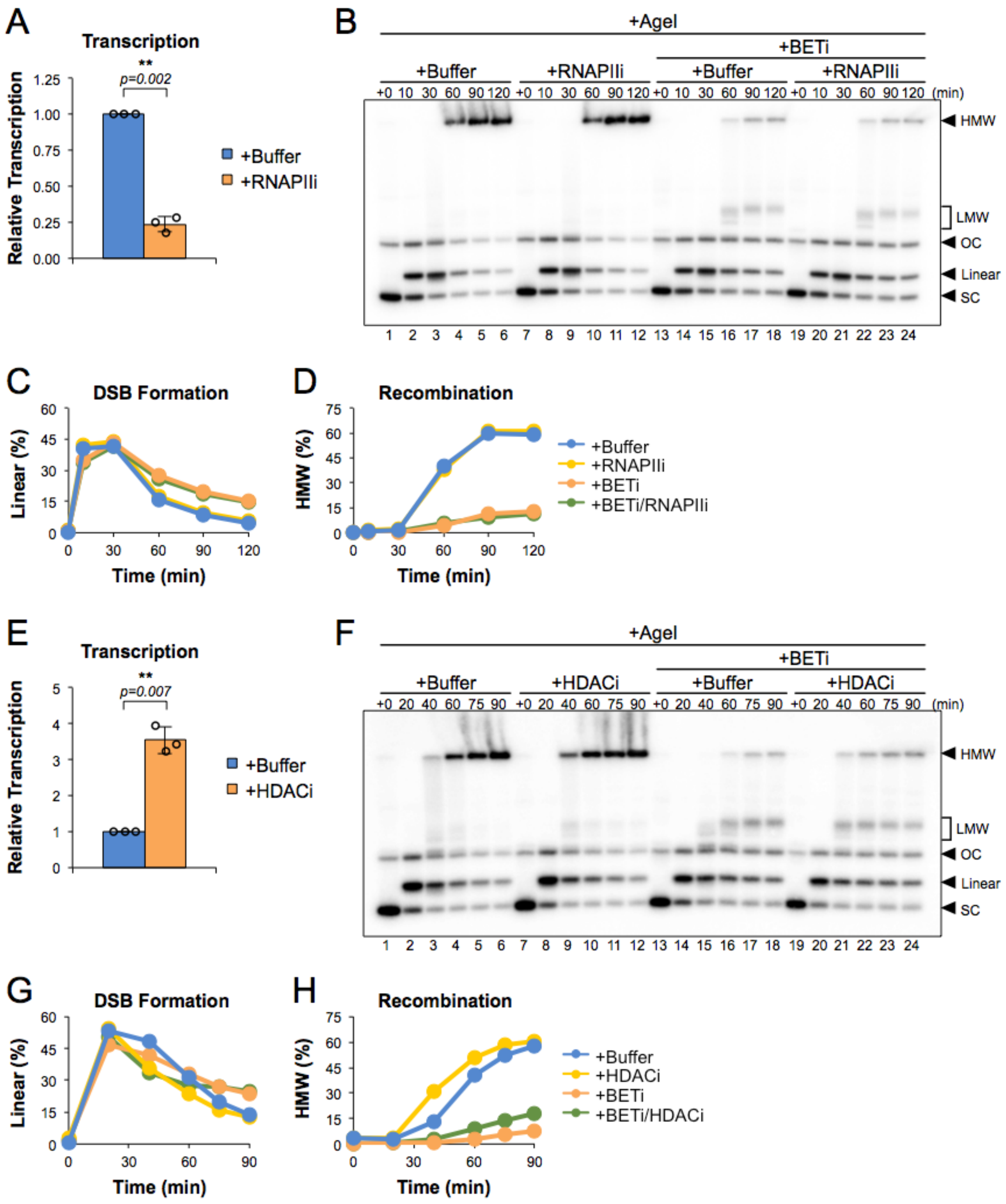

Figure S6. Chromatin signaling stimulates homology-directed repair, Related to Figure 2. (**A**) pDSB was replicated in extract supplemented with buffer or α-amanitin (RNAPIIi). After 120 minutes, RNA was isolated and analyzed by RT-qPCR (n=3 independent experiments). (**B**) pDSB was replicated with α^{32}P[dATP] in reactions containing buffer, RNAPIIi, JQ1 (BETi), or a combination of RNAPIIi and BETi. After 45 minutes, AgeI was added and samples were withdrawn at the indicated time points for 1D gel electrophoresis (n=2 independent experiments). (**C-D**) Quantitation of linear *(C)* and HMW *(D)* molecules from *(B)*. (**E**) pDSB was replicated in extract supplemented with buffer or Vorinostat (HDACi). After 120 minutes, RNA was isolated and analyzed by RT-qPCR (n=3 independent experiments). (**F**) pDSB was replicated with α^{32}P[dATP] in reactions containing buffer, HDACi, BETi, or a combination of HDACi and BETi. After 45 minutes, AgeI was added and samples were withdrawn at the indicated time points for 1D gel electrophoresis (n=2 independent experiments). (**G-H**) Quantitation of linear *(G)* and HMW *(H)* molecules from *(F)*. Error bars represent +/- one standard deviation from the mean. Student two-tailed t-test: p-value < 0.01 (**).

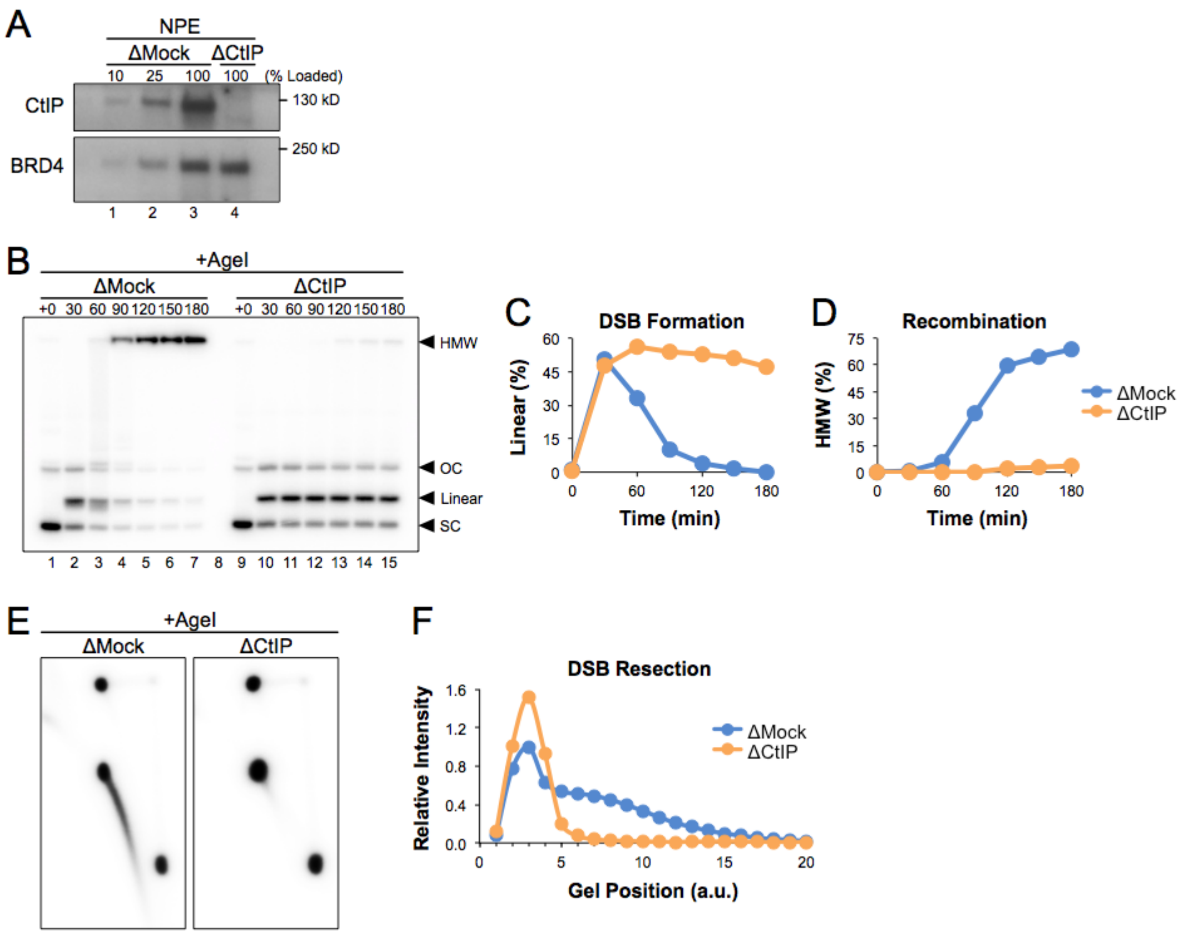

Figure S7. CtIP is essential for resection and homology-directed repair, Related to Figure 2. (**A**) Mock-depleted (ΔMock) or CtIP-depleted (ΔCtIP) NPE was analyzed by Western blot using the indicated antibodies (n=2 independent experiments). (**B**) pDSB was replicated with α^{32}P[dATP] in mock- or CtIP-depleted extracts. After 45 minutes, reactions were supplemented with AgeI and samples were withdrawn at the indicated time points for 1D gel electrophoresis (n=2 independent experiments). (**C-D**) Quantitation of linear *(C)* and HMW *(D)* molecules from *(B)*. (**E**) pDSB was replicated with α^{32}P[dATP] in mock- or CtIP-depleted extracts. After 45 minutes, reactions were supplemented with AgeI and samples were withdrawn 30 minutes later for 2D gel electrophoresis (n=2 independent experiments). (**F**) Quantitation of linear and resected molecules in *(E)*.

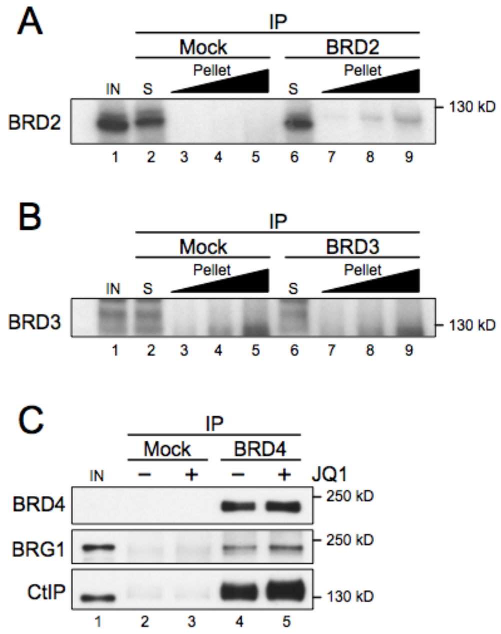

Figure S8. Immunoprecipitation, Related to Figure 4. (**A-B**) A mixture of HSS and NPE was immunoprecipitated with beads conjugated to mock, BRD2, or BRD3 antibodies. Input (IN), supernatant (S) and bead-bound (Pellet) proteins were analyzed by Western blot with the indicated antibodies (n=2 independent experiments). (**C**) A mixture of HSS and NPE supplemented with buffer or JQ1 was immunoprecipitated with beads conjugated to mock or BRD4 antibodies. Bead-bound proteins were analyzed by Western blot with the indicated antibodies (n=2 independent experiments).

Supplemental References

1. Long, D.T., Räschle, M., Joukov, V. & Walter, J.C. Mechanism of RAD51-dependent DNA interstrand cross-link repair. *Science* **333**, 84-7 (2011).
2. Chen, C.F., Chen, P.L., Zhong, Q., Sharp, Z.D. & Lee, W.H. Expression of BRC repeats in breast cancer cells disrupts the BRCA2-Rad51 complex and leads to radiation hypersensitivity and loss of G(2)/M checkpoint control. *J Biol Chem* **274**, 32931-5 (1999).
3. Chen, P.L. et al. The BRC repeats in BRCA2 are critical for RAD51 binding and resistance to methyl methanesulfonate treatment. *Proc Natl Acad Sci U S A* **95**, 5287-92 (1998).
4. Nomme, J. et al. Design of potent inhibitors of human RAD51 recombinase based on BRC motifs of BRCA2 protein: modeling and experimental validation of a chimera peptide. *J Med Chem* **53**, 5782-91 (2010).
5. Rajendra, E. & Venkitaraman, A.R. Two modules in the BRC repeats of BRCA2 mediate structural and functional interactions with the RAD51 recombinase. *Nucleic Acids Res* **38**, 82-96 (2010).
6. Amunugama, R. et al. Replication Fork Reversal during DNA Interstrand Crosslink Repair Requires CMG Unloading. *Cell Rep* **23**, 3419-3428 (2018).
