# Supplementary material for: BRD4 promotes resection and homology-directed repair of DNA double-strand breaks
Source: Nat Commun. 2022 May 31;13:3016. doi: 10.1038/s41467-022-30787-6 (PMC9156784; doi:10.1038/s41467-022-30787-6)
Supplement: Supplementary file 2 — Reporting Summary [file 41467_2022_30787_MOESM2_ESM.pdf]

nature portfolio

Corresponding author(s): David T. Long

Last updated by author(s): May 9, 2022

Reporting Summary

Nature Portfolio wishes to improve the reproducibility of the work that we publish. This form provides structure for consistency and transparency in reporting. For further information on Nature Portfolio policies, see our Editorial Policies and the Editorial Policy Checklist.

Statistics

For all statistical analyses, confirm that the following items are present in the figure legend, table legend, main text, or Methods section.

n/a	Confirmed	
☐	☒	The exact sample size (*n*) for each experimental group/condition, given as a discrete number and unit of measurement
☒	☐	A statement on whether measurements were taken from distinct samples or whether the same sample was measured repeatedly
☐	☒	The statistical test(s) used AND whether they are one- or two-sided<br>*Only common tests should be described solely by name; describe more complex techniques in the Methods section.*
☒	☐	A description of all covariates tested
☒	☐	A description of any assumptions or corrections, such as tests of normality and adjustment for multiple comparisons
☐	☒	A full description of the statistical parameters including central tendency (e.g. means) or other basic estimates (e.g. regression coefficient) AND variation (e.g. standard deviation) or associated estimates of uncertainty (e.g. confidence intervals)
☐	☒	For null hypothesis testing, the test statistic (e.g. *F*, *t*, *r*) with confidence intervals, effect sizes, degrees of freedom and *P* value noted<br>*Give P values as exact values whenever suitable.*
☒	☐	For Bayesian analysis, information on the choice of priors and Markov chain Monte Carlo settings
☒	☐	For hierarchical and complex designs, identification of the appropriate level for tests and full reporting of outcomes
☒	☐	Estimates of effect sizes (e.g. Cohen's *d*, Pearson's *r*), indicating how they were calculated

Our web collection on statistics for biologists contains articles on many of the points above.

Software and code

Policy information about availability of computer code

Data collection	Amplicon sequencing was performed by Genewiz (http://genewiz.com), who performed variant detection analysis for SNPs and INDELs up to 20bp in length.
Data analysis	Student t-tests were performed using Microsoft Excel (v14.7.7).

For manuscripts utilizing custom algorithms or software that are central to the research but not yet described in published literature, software must be made available to editors and reviewers. We strongly encourage code deposition in a community repository (e.g. GitHub). See the Nature Portfolio guidelines for submitting code & software for further information.

Data

Policy information about availability of data

All manuscripts must include a data availability statement. This statement should provide the following information, where applicable:

- Accession codes, unique identifiers, or web links for publicly available datasets
- A description of any restrictions on data availability
- For clinical datasets or third party data, please ensure that the statement adheres to our policy

The sequencing data generated in this study are publicly available through the Harvard Dataverse Repository, https://doi.org/10.7910/DVN/CEFLQ8. Uncropped Western blot images are provided in the Source Data file.

Field-specific reporting

Please select the one below that is the best fit for your research. If you are not sure, read the appropriate sections before making your selection.

☒ Life sciences ☐ Behavioural & social sciences ☐ Ecological, evolutionary & environmental sciences

For a reference copy of the document with all sections, see nature.com/documents/nr-reporting-summary-flat.pdf

Life sciences study design

All studies must disclose on these points even when the disclosure is negative.

Sample size	All experiments were performed at least twice and significance was determined using student t-tests.
Data exclusions	No data were excluded from analysis.
Replication	All experiments were performed successfully at least twice and where indicated, representative data are shown (e.g. gel images).
Randomization	Not applicable. Animals were not used in groups for experimentation.
Blinding	Not applicable. Animals were not used in groups for experimentation.

Reporting for specific materials, systems and methods

We require information from authors about some types of materials, experimental systems and methods used in many studies. Here, indicate whether each material, system or method listed is relevant to your study. If you are not sure if a list item applies to your research, read the appropriate section before selecting a response.

Materials & experimental systems

n/a	Involved in the study	
☐	☒	Antibodies
☒	☐	Eukaryotic cell lines
☒	☐	Palaeontology and archaeology
☐	☒	Animals and other organisms
☒	☐	Human research participants
☒	☐	Clinical data
☒	☐	Dual use research of concern

Methods

n/a	Involved in the study	
☒	☐	ChIP-seq
☒	☐	Flow cytometry
☒	☐	MRI-based neuroimaging

Antibodies

Antibodies used

Commercial antibodies were used to detect phosphorylated Chk1 (Cell Signaling, #2341, 1:1000 dilution), Chk1 (Bethyl Laboratories, A300-298A, histone H3 (Thermo Fisher, PA5-16183, 1:4,000 dilution), and BRG1 (Bethyl Laboratories, A300-813A, 1:4,000 dilution). Xenopus laevis BRD2, BRD3, and BRD4 antibodies were produced by New England Peptide (NEP) using the following antigen sequences: BRD2-KPHDKAESAHQVSVT, BRD3-EPRRERYKGATQAS, and BRD4-NFQSELMEIFEQNLFS and used at 1:4,000 dilution. Xenopus laevis Mre11 and CtIP antibodies were generously provided by the laboratories of Jean Gautier and Richard Baer from Columbia University and used at 1:4,000 dilution. Xenopus laevis RAD51 and RPA antibodies were developed previously and used at 1:4,000 dilution.

Validation

Histone H3 - Validated for Xenopus by Thermo Fisher and described in Barrows and Long, Cell-free transcription in Xenopus egg extract. J Biol Chem. 294(51), 19645-19654 (2019).

Chk1 - Provided by Bethyl laboratories and chosen based on high sequence homology between the antigen (residues 250 and 300) and Xenopus Chk1 protein.

BRG1 - As described in Miyamoto et al, Nuclear actin polymerization is required for transcriptional reprogramming of Oct4 by oocytes. Genes Dev 25(9), 946-958 (2011).

pChk1 - As described in Long et al, BRCA1 promotes unloading of the CMG helicase from a stalled DNA replication fork. Mol Cell 56(1), 174-185 (2014).

Mre11 - As described in Di Virgilio, M. & Gautier, J. Repair of double-strand breaks by nonhomologous end joining in the absence of Mre11. J Cell Biol 171, 765-71 (2005).

CtIP - As described in Peterson, S.E. et al. Cdk1 uncouples CtIP-dependent resection and Rad51 filament formation during M-phase double-strand break repair. J Cell Biol 194, 705-20 (2011).

RAD51 - As described in Long, D.T., Räschle, M., Joukov, V. & Walter, J.C. Mechanism of RAD51-dependent DNA interstrand cross-link repair. Science 333, 84-7 (2011).

RPA - As described in Fang, F. & Newport, J.W. Distinct roles of cdk2 and cdc2 in RP-A phosphorylation during the cell cycle. J Cell Sci 106 (Pt 3), 983-94 (1993).

BRD2, 3, 4 - Primary antibodies were validated by New England Peptide (NEP) for recognition of the antigen sequence by ELISA, providing a relative measurement of antigen specific antibody present in the serum samples. The serum is diluted until the antibody levels approach background levels. The titer is then determined to be that dilution which gives a reading approximately 3x above background. Experimentally, each antibody was validated by Western blotting for recognition of proteins based on predicted size, followed by immunoprecipitation to confirm specific interaction with the target protein.

Animals and other organisms

Policy information about studies involving animals; ARRIVE guidelines recommended for reporting animal research

Laboratory animals	"Wild type" Xenopus laevis (African clawed frogs) were provided by Nasco. Male frogs (>12 months) were used for isolation of sperm chromatin and female frogs (>12 months) were used for egg laying.
Wild animals	The study did not involve wild animals.
Field-collected samples	The study did not involve field-collected samples.
Ethics oversight	The use of vertebrate animals for this study complies with all institutional and federal regulations. Veterinary care at the Medical University of South Carolina is provided by the Division of Laboratory Animal Resources (DLAR) in conjunction with the Association for Assessment and Accreditation of Laboratory Animal Care International (AAALAC International) -accredited Institutional Animal Care and Use Committee (IACUC).

Note that full information on the approval of the study protocol must also be provided in the manuscript.
